# The Importance of Signal Strength in Quantitative Assessment of Retinal Vessel Density Using Optical Coherence Tomography Angiography

**DOI:** 10.1038/s41598-018-31321-9

**Published:** 2018-08-27

**Authors:** Hyung Bin Lim, Yong Woo Kim, Ju Mi Kim, Young Joon Jo, Jung Yeul Kim

**Affiliations:** 1 Department of Ophthalmology, Chungnam National University College of Medicine, Daejeon, Republic of Korea; 2 Department of Ophthalmology, Armed Forces Capital Hospital, Seongnam, Republic of Korea; 3 Department of Ophthalmology, Seoul National University Hospital, Seoul, Republic of Korea

## Abstract

The quality of the scan image is important in microvascular circulation analysis using optical coherence tomography (OCTA). We aimed to investigate the effect of signal strength (SS) on OCTA metrics and minimum SS level that could be considered optimal. Macular 6 × 6 mm angiography images were acquired, and all subjects were divided into four groups according to the SS (SS 7, SS 8, SS 9, and SS 10) of the OCTA image. Vessel density (VD), perfusion density (PD), and foveal avascular zone (FAZ) metrics of the superficial capillary plexus were compared. In total, 446 eyes from young healthy subjects were included. As the SS increased from 7 to 10, the VD and PD of the total area, and the FAZ area increased significantly (all, p < 0.001), but there were no significant difference between SS 9 and SS 10 in all metrics. Correlation analysis between the SS and each parameter showed a high correlation coefficient (VD, r = 0.668; PD, r = 0.671; FAZ area, r = 0.570; all, p < 0.001). The measurements of VD, PD, and FAZ using OCTA varied significantly with the SS, and at least 9 of SS is recommended. The physician should be careful in the analyses of OCTA measurements showing different values of the SS.

## Introduction

Since fluorescein angiography (FA) was introduced by Novotny and Alvis in the 1960s^1^, it has been considered the gold standard method for imaging of retinal vessels. FA provides functional information regarding dye leakage, information on vascular permeability, and the ability to stain structures such as chorioretinal scars or drusen. However, FA is invasive, takes a relatively long time to obtain a vascular image, and has side effects, such as urticaria and anaphylactic shock. In addition, FA cannot differentiate and visualize the vasculature of various retinal and choroidal layers.

The recently introduced technique of optical coherence tomography angiography (OCTA) provides fast and noninvasive assessment of the capillary network. OCTA differentiates and visualizes the microvasculature of various retinal and choroidal layers at different depths. The Angioplex from the Zeiss Cirrus 5000 (Carl Zeiss Meditec, Dublin, CA, USA) is a commercially available OCTA instrument. A new software update recently released for the Angioplex system allowed quantification of the vessel density (VD), perfusion density (PD), and foveal avascular zone (FAZ) area in the superficial capillary plexus (SCP).

Quantitative assessment of the retinal VD and FAZ area is a potential biomarker for macular ischemia in diabetic retinopathy (DR) and other retinal vascular diseases^2,3^. The assessment of the retinal VD and FAZ areas may be useful for monitoring and detecting retinal vascular diseases, including DR and retinal vascular occlusion. In recent studies, normative ranges of vessel densities^4–6^ and FAZ areas^7^ measured with OCTA have been reported. However, various factors can influence OCTA measurements, and consideration of these factors is necessary for correct OCTA data analyses.

Inclusion criteria for the acceptable signal strength (SS) has varied widely in previous reports. In time domain OCT, each OCT scan is indicated with an SS parameter, which ranges from 0 to 10. SS values of 6^8,9^ and 7^10,11^ have been used as the minimal inclusion criteria in various studies. For the Cirrus High Definition SD-OCT (Carl Zeiss Meditec), the manufacturer defined scans of adequate quality as an SS ≥ 6^12^. The SS is a useful index that reflects OCT image quality, and the SS has been significantly correlated with repeatability and reproducibility of OCT measurements^13,14^. Although several previous studies using OCTA have mentioned the effects of SS, there has been no consensus on a minimum SS level that could be considered optimal. This study therefore investigated the effect of SS on OCTA measurements.

## Results

### Patient characteristics

In total, 292 eyes of males and 154 eyes of females were included. The subjects grouped by the SS were composed of 94 (SS 7), 162 (SS 8), 125 (SS 9), and 65 (SS 10) eyes. The mean ages of the SS 7, 8, 9, and 10 groups were 26.12 ± 8.53, 25.95 ± 8.08, 27.15 ± 9.05, and 27.32 ± 7.76 years, respectively. No statistically significant differences were found in age and sex among the four groups. There was no significant difference among the four groups in ocular characteristics, including the BCVA and spherical equivalent, IOP, central corneal thickness, axial length, CFT, and average RNFL thickness. The baseline characteristics of the participants are shown in Table 1.

**Table 1:** Demographics and clinical characteristics of the subjects.

	SS 7 (n = 94)	SS 8 (n = 162)	SS 9 (n = 125)	SS 10 (n = 65)	p-value
Age (mean ± SD, years)	26.12 ± 8.53	25.95 ± 8.08	27.15 ± 9.05	27.32 ± 7.76	0.526^*^
Sex (male/female)	60/34	107/55	87/38	38/27	0.477^†^
BCVA (mean ± SD, LogMAR)	0.02 ± 0.05	0.02 ± 0.05	0.02 ± 0.05	0.03 ± 0.08	0.587^*^
Spherical equivalent (mean ± SD, diopters)	−2.44 ± 2.25	−1.95 ± 2.30	−1.90 ± 2.36	−2.18 ± 1.90	0.099^*^
Intraocular pressure (mean ± SD, mmHg)	15.77 ± 3.57	15.86 ± 3.45	15.66 ± 3.62	15.92 ± 2.76	0.950^*^
Central corneal thickness (mean ± SD, μm)	537.93 ± 45.76	545.00 ± 50.64	541.74 ± 40.69	553.93 ± 48.39	0.219^*^
Axial length (mean ± SD, mm)	25.52 ± 1.26	25.17 ± 1.27	24.95 ± 1.26	25.24 ± 1.05	0.101^*^
Central macular thickness (mean ± SD, μm)	251.09 ± 19.43	253.07 ± 19.57	255.08 ± 20.99	250.69 ± 19.27	0.379^*^
Average RNFL thickness (mean ± SD, μm)	94.90 ± 8.71	95.70 ± 8.72	96.81 ± 8.59	98.63 ± 8.38	0.071^*^

SS = signal strength; BCVA = best-corrected visual acuity; logMAR = logarithm of the minimum angle of resolutions; RNFL = retinal nerve fiber layer.

^*^The *p*-value was obtained from one-way analysis of variance.

^†^The *p*-value was obtained from the chi-squared test.

### Vessel density

**Figure 1:**
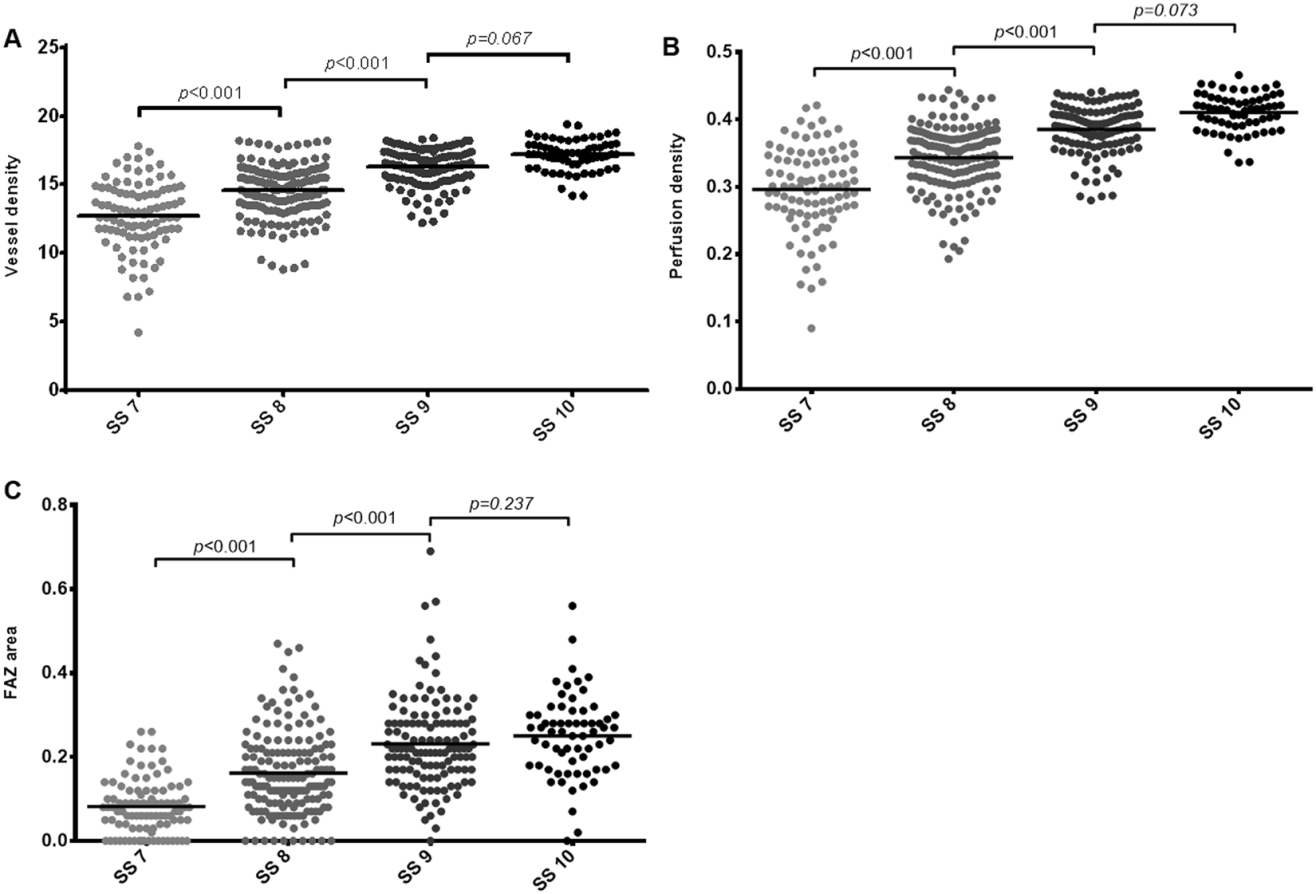
Scatter plots showing the significant relationships between signal strength and vessel density for the total area **(A)**, perfusion density for the total area **(B)**, and the foveal avascular zone area **(C)**. The p-values were calculated using the Student’s *t*-test.

As the SS increased from 7 to 10, the VD measurements in all areas tended to increase; these differences were statistically significant (all, p < 0.001). In post-hoc analyses, there were significant differences among the SS 7, SS 8, and SS 9 groups in all parameters (all, p < 0.001). The VD of the OuterSuperior (p = 0.022), OuterInferior (p = 0.044), and OuterTemporal (p < 0.001) regions were significantly higher in the SS 10 group than in the SS 9 group; there were no significant difference between the SS 9 and SS 10 groups in any other region (all, p > 0.05) (Table 2; Fig. 1A).

**Table 2:** Comparisons of the vessel density among groups with a signal strength (SS) from 7 to 10 (mm^−1^).

	SS 7 (n = 94)	SS 8 (n = 162)	SS 9 (n = 125)	SS 10 (n = 65)	p-value^*^	p-value^†^	p-value^‡^	p-value^§^
Total area	12.71 ± 2.53	14.56 ± 1.91	16.18 ± 1.53	17.17 ± 1.12	**<0**.**001**	**<0**.**001**	**<0**.**001**	0.067
Center	3.98 ± 2.62	5.45 ± 2.84	6.85 ± 2.61	7.66 ± 2.42	**<0**.**001**	**<0**.**001**	**<0**.**001**	0.289
InnerAverage	11.66 ± 3.41	13.51 ± 2.94	15.66 ± 2.16	16.64 ± 1.60	**<0**.**001**	**<0**.**001**	**<0**.**001**	0.172
InnerSuperior	12.69 ± 3.47	14.73 ± 2.92	16.50 ± 2.22	17.37 ± 1.46	**<0**.**001**	**<0**.**001**	**<0**.**001**	0.214
InnerNasal	11.60 ± 3.96	13.75 ± 3.25	15.74 ± 2.44	16.67 ± 1.72	**<0**.**001**	**<0**.**001**	**<0**.**001**	0.285
InnerInferior	12.29 ± 3.61	13.92 ± 3.13	16.06 ± 2.35	16.89 ± 1.84	**<0**.**001**	**<0**.**001**	**<0**.**001**	0.379
InnerTemporal	9.62 ± 3.86	11.81 ± 3.82	14.30 ± 2.97	15.63 ± 2.29	**<0**.**001**	**<0**.**001**	**<0**.**001**	0.069
OuterAverage	14.65 ± 2.38	15.18 ± 1.74	16.68 ± 1.44	17.68 ± 1.05	**<0**.**001**	**<0**.**001**	**<0**.**001**	0.084
OuterSuperior	14.54 ± 2.63	16.13 ± 1.86	17.41 ± 1.52	18.25 ± 1.00	**<0**.**001**	**<0**.**001**	**<0**.**001**	**0**.**022**
OuterNasal	16.67 ± 3.04	18.21 ± 2.05	19.20 ± 1.43	19.63 ± 1.00	**<0**.**001**	**<0**.**001**	**<0**.**001**	0.999
OuterInferior	14.06 ± 2.64	15.76 ± 2.08	17.21 ± 1.81	18.07 ± 1.47	**<0**.**001**	**<0**.**001**	**<0**.**001**	**0**.**044**
OuterTemporal	8.27 ± 3.67	10.55 ± 3.24	12.86 ± 2.68	14.78 ± 2.19	**<0**.**001**	**<0**.**001**	**<0**.**001**	**<0**.**001**

Mean ± standard deviation.

^*^The *p*-value was obtained from one-way analysis of variance.

^†^The *p*-value was obtained from the post-hoc test (Bonferroni) between the SS 7 and SS 8 groups.

^‡^The *p*-value was obtained from the post-hoc test (Bonferroni) between the SS 8 and SS 9 groups.

^§^The *p*-value was obtained from the post-hoc test (Bonferroni) between the SS 9 and SS 10 groups.

Boldface numbers indicate statistically significant differences at p < 0.05.

### Perfusion density

In the same manner as the VD, the PD measurements in all areas significantly increased as the SS increased from 7 to 10. In post-hoc analyses, the PD showed the same patterns as the VD in all parameters. The PD of the OuterSuperior (p = 0.020), OuterInferior (p = 0.018), and OuterTemporal (p < 0.001) regions were significantly higher in the SS 10 group than in the SS 9 group; there were no significant differences between the SS 9 and SS 10 groups in any other parameters (all, p > 0.05) (Table 3; Fig. 1B).

**Table 3:** Comparisons of perfusion densities among groups with a signal strength (SS) from 7 to 10.

	SS 7 (n = 94)	SS 8 (n = 162)	SS 9 (n = 125)	SS 10 (n = 65)	p-value^*^	p-value^†^	p-value^‡^	p-value^§^
Total area	0.296 ± 0.063	0.343 ± 0.048	0.384 ± 0.039	0.410 ± 0.028	**<0.001**	**<0.001**	**<0.001**	0.073
Center	0.082 ± 0.057	0.115 ± 0.063	0.146 ± 0.058	0.165 ± 0.056	**<0.001**	**<0.001**	**<0.001**	0.234
InnerAverage	0.260 ± 0.080	0.310 ± 0.070	0.360 ± 0.053	0.385 ± 0.040	**<0.001**	**<0.001**	**<0.001**	0.125
InnerSuperior	0.291 ± 0.085	0.343 ± 0.072	0.386 ± 0.055	0.409 ± 0.037	**<0.001**	**<0.001**	**<0.001**	0.185
InnerNasal	0.259 ± 0.094	0.311 ± 0.080	0.358 ± 0.059	0.381 ± 0.043	**<0.001**	**<0.001**	**<0.001**	0.278
InnerInferior	0.280 ± 0.087	0.322 ± 0.077	0.376 ± 0.059	0.394 ± 0.047	**<0.001**	**<0.001**	**<0.001**	0.530
InnerTemporal	0.212 ± 0.090	0.265 ± 0.088	0.324 ± 0.071	0.356 ± 0.055	**<0.001**	**<0.001**	**<0.001**	0.055
OuterAverage	0.315 ± 0.063	0.362 ± 0.055	0.400 ± 0.037	0.427 ± 0.027	**<0.001**	**<0.001**	**<0.001**	0.091
OuterSuperior	0.345 ± 0.067	0.387 ± 0.049	0.422 ± 0.040	0.444 ± 0.025	**<0.001**	**<0.001**	**<0.001**	**0**.**020**
OuterNasal	0.391 ± 0.083	0.435 ± 0.058	0.462 ± 0.039	0.475 ± 0.027	**<0.001**	**<0.001**	**<0.001**	0.703
OuterInferior	0.332 ± 0.068	0.377 ± 0.055	0.416 ± 0.048	0.440 ± 0.039	**<0.001**	**<0.001**	**<0.001**	**0**.**018**
OuterTemporal	0.191 ± 0.088	0.246 ± 0.077	0.300 ± 0.065	0.349 ± 0.054	**<0.001**	**<0.001**	**<0.001**	**<0**.**001**

Mean ± standard deviation.

^*^The *p*-value was obtained from one-way analysis of variance.

^†^The *p*-value was obtained from the post-hoc test (Bonferroni) between the SS 7 and SS 8 groups.

^‡^The *p*-value was obtained from the post-hoc test (Bonferroni) between the SS 8 and SS 9 groups.

^§^The *p*-value was obtained from the post-hoc test (Bonferroni) between the SS 9 and SS 10 groups.

Boldface numbers indicate statistically significant differences at p < 0.05.

### Foveal avascular zone

As the SS increased from 7 to 10, all FAZ metrics increased significantly (all, p < 0.001). In post-hoc analyses, the area and perimeter of the FAZ differed significantly among the SS 7, SS 8, and SS 9 groups (all, p < 0.001). There were no significant differences between the SS 9 and SS 10 groups in the area (p = 0.237) and perimeter (p = 0.324) of the FAZ. The circularity analyses showed that the circularity of the SS 8 group was significantly higher than that of the SS 7 group (p < 0.001). However, there were no significant differences among the SS 8, 9, and 10 groups (all, p > 0.05) (Table 4; Fig. 1C).

**Table 4:** Comparisons of foveal avascular area metrics among groups with a signal strength (SS) from 7 to 10.

	SS 7 (n = 94)	SS 8 (n = 162)	SS 9 (n = 125)	SS 10 (n = 65)	p-value^*^	p-value^†^	p-value^‡^	p-value^§^
Area (mean ± SD, mm^2^)	0.082 ± 0.066	0.161 ± 0.100	0.231 ± 0.107	0.250 ± 0.097	** < 0**.**001**	** < 0**.**001**	** < 0**.**001**	0.237
Perimeter (mean ± SD, mm)	1.095 ± 0.724	1.655 ± 0.648	2.072 ± 0.654	2.081 ± 0.541	** < 0**.**001**	** < 0**.**001**	** < 0**.**001**	0.324
Circularity (mean ± SD)	0.508 ± 0.276	0.616 ± 0.183	0.666 ± 0.131	0.689 ± 0.126	** < 0**.**001**	** < 0**.**001**	0.319	0.535

Mean ± standard deviation.

^*^The *p*-value was obtained from the one-way analysis of variance.

^†^The *p*-value was obtained from the post-hoc test (Bonferroni) between the SS 7 and SS 8 groups.

^‡^The *p*-value was obtained from the post-hoc test (Bonferroni) between the SS 8 and SS 9 groups.

^§^The *p*-value was obtained from the post-hoc test (Bonferroni) between the SS 9 and SS 10 groups.

Boldface numbers indicate statistically significant differences at p < 0.05.

### Correlation analyses between clinical factors and OCTA measurements

The correlation analyses showed that the VD and PD for total areas had a very high positive correlation with the SS, which was statistically significant (VD: r = 0.668, p < 0.001; PD: r = 0.671, p < 0.001). FAZ metrics also showed high positive correlation coefficients with the SS (area: r = 0.570, p < 0.001; perimeter: r = 0.549, p < 0.001; circularity: r = 0.430, p < 0.001). In addition, the CFT showed a significant correlation with OCTA measurements, but the correlation coefficient was low (VD: r = 0.115, p = 0.013; PD: r = 0.113, p = 0.015; FAZ area: r = −0.139, p = 0.003; FAZ perimeter: r = −0.117, p = 0.012). Other factors including age, BCVA, IOP, spherical equivalent, central corneal thickness, and axial length did not have significant correlations (all, p > 0.05) (Table 5).

**Table 5:** Pearson’s correlation coefficients between various clinical factors and optical coherence tomography angiography measurements.

	Vessel density		Perfusion density		FAZ area		FAZ perimeter		FAZ circularity	
	r	p-value	r	p-value	r	p-value	r	p-value	r	p-value
Age	0.050	0.285	0.040	0.393	0.018	0.697	0.028	0.540	−0.018	0.703
BCVA (LogMAR)	0.014	0.764	0.014	0.757	0.016	0.723	0.022	0.637	0.059	0.203
IOP	−0.059	0.208	−0.048	0.298	−0.006	0.905	−0.015	0.747	−0.028	0.540
SE	0.032	0.490	0.034	0.468	0.048	0.298	0.038	0.414	−0.032	0.494
CCT	−0.014	0.766	0.000	0.999	0.040	0.406	0.037	0.442	0.003	0.959
Axial length	−0.046	0.320	−0.046	0.326	−0.059	0.206	−0.047	0.317	0.019	0.685
CFT	**0**.**115**	**0**.**013**	**0**.**113**	**0**.**015**	**−0**.**139**	**0**.**003**	**−0**.**117**	**0**.**012**	−0.005	0.908
Signal strength	**0**.**668**	** < 0**.**001**	**0**.**671**	** < 0**.**001**	**0**.**570**	** < 0**.**001**	**0**.**549**	** < 0**.**001**	**0**.**430**	** < 0**.**001**

FAZ = foveal avascular zone; BCVA = best-corrected visual acuity; IOP = intraocular pressure; SE = spherical equivalent; CCT = central corneal thickness; CFT = central foveal thickness.

Boldface numbers indicate statistically significant differences at p < 0.05.

### Comparisons of two consecutive angiography scans showing different signal strength in the same subjects

**Figure 2:**
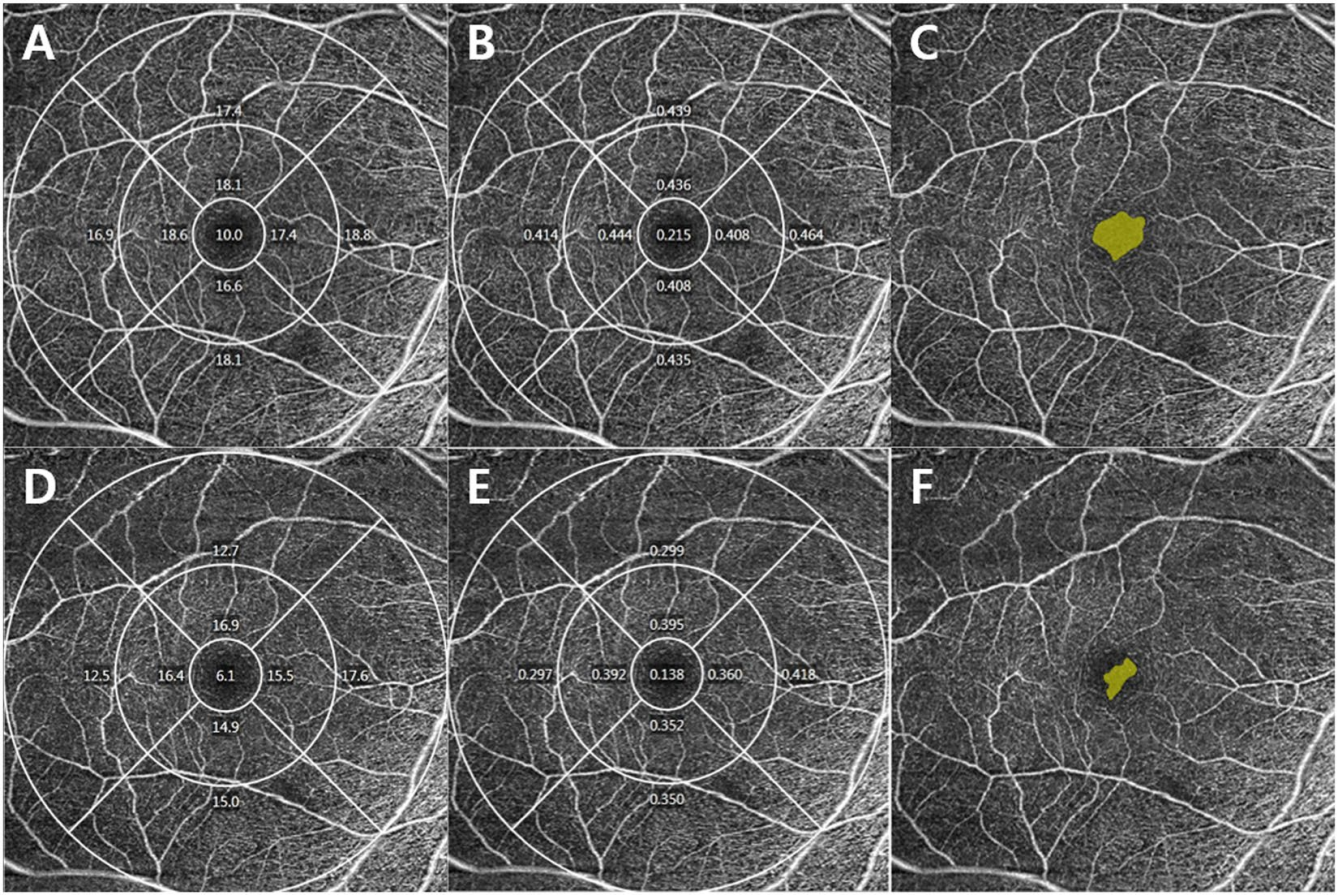
Optical coherence tomography angiography images of signal strength (SS) 10 (upper row) and 8 (lower row) in the same subject. A vessel density **(A**,**D)** and perfusion density **(B**,**E)** map of the superficial capillary plexus according to the Early Treatment of Diabetic Retinopathy Study subfields. The automatically detected foveal avascular zone (FAZ). The FAZ areas were 0.29 mm2 in SS 10 image **(C)** and 0.11 mm2 in SS 8 image **(F)**.

In addition to 446 eyes included in this study, additional analysis in the OCTA scans showing different signal strength in the same subjects (17 eyes) were performed. These two scans were obtained consecutively. The VD and PD measurements in SS 9 scans were significantly higher than SS 8 scans (all p < 0.05). The FAZ (p = 0.042) area and circularity (p = 0.046) were also higher in SS 9 than SS 8. There were no significant differences in the FAZ perimeter (p = 0.118) (Table 6). In addition, the VD, PD, and FAZ area measurements of two consecutive scans showing SS 10 and 8 in the same subject are presented in Fig. 2.

**Table 6:** Comparisons of the vascular density, perfusion density, and foveal avascular zone metrics between two consecutive macular angiography scans showing different signal strength in the same subjects (n = 17).

	1^st^ measurements Signal strength = 8	2^nd^ measurements Signal strength = 9	p-value
Vessel density			
Total area (mean ± SD, mm^−1^)	15.31 ± 2.13	16.64 ± 1.29	** < 0**.**001**
Center (mean ± SD, mm^−1^)	6.87 ± 3.05	7.91 ± 2.32	**0**.**031**
Inner - average (mean ± SD, mm^−1^)	14.99 ± 2.63	16.31 ± 2.22	**0**.**010**
Outer - average (mean ± SD, mm^−1^)	15.72 ± 2.09	17.06 ± 1.20	** < 0**.**001**
Perfusion density			
Total area (mean ± SD)	0.380 ± 0.054	0.414 ± 0.033	**0**.**001**
Center (mean ± SD)	0.149 ± 0.072	0.173 ± 0.054	**0**.**025**
Inner - average (mean ± SD)	0.349 ± 0.069	0.382 ± 0.058	**0**.**012**
Outer - average (mean ± SD)	0.380 ± 0.054	0.414 ± 0.033	** < 0**.**001**
Foveal avascular area			
Area (mean ± SD, mm^2^)	0.18 ± 0.09	0.22 ± 0.10	**0**.**042**
Perimeter (mean ± SD, mm)	1.83 ± 0.42	1.91 ± 0.41	0.118
Circularity (mean ± SD)	0.67 ± 0.09	0.72 ± 0.08	**0**.**046**

SD, standard deviation.

The p-value was obtained using a Wilcoxon signed rank test.

Boldface numbers indicate statistically significant differences at p < 0.05.

## Discussion

Several recent studies have evaluated the use of OCTA for various diseases, including DR, retinal vein occlusion, retinal artery occlusion, age-related macular degeneration, and glaucoma, and analyzing the findings of VD and FAZ metrics in each disease. In DR, previous studies reported that the FAZ area of DR patients increased significantly when correlated with their DR severities^15,16^. This finding was similar to those reported by other investigators using FA^3,17^. In addition, a few studies assessed the VD in patients with DR, and reported that the vessel and skeleton densities showed a negative correlation with DR severities^2^. Furthermore, Durbin *et al*. reported that the VD measured by OCTA showed good diagnostic power for DR using a receiver operating characteristic curve^18^.

In the present study, it was confirmed that the SS strongly influenced the OCTA measurements. As the SS increased from 7 to 9, OCTA measurements increased steeply. When the SS increased from 9 to 10, the measured values also increased, but there was no significant difference between the two groups in many parameters. In the FAZ analyses of the SS 7 and SS 8 groups, a detection error was identified in many subjects, which made accurate FAZ analyses impossible (Fig. 2F). In addition, the angiography scans, which were performed twice consecutively in the same subjects, showed significantly difference. In the correlation coefficient analyses between various clinical factors and OCTA measurements, the SS showed high positive correlation coefficients from 0.430 to 0.671, which were significantly higher than other clinical factors.

The scan image quality is very important for accurate analyses of the imaging modality. In clinical practice, the OCT scan image quality is influenced by many factors, such as examiner skill, patient cooperation, and media opacity. Images with a higher SS yield better clarity and improved segmentation, resulting in improved repeatability. In addition, previous studies reported that quantitative metrics could be affected by the SS^13,14^. If an acceptable SS is not obtained, the repeatability and reproducibility are decreased and the test result becomes unreliable. For accurate data analyses, recommended SS values should be obtained. For example, the SS of the Cirrus HD-OCT should be ≥6, the Spectralis OCT (Heidelberg Engineering, Heidelberg, Germany) recommends values of more than 15 in Q score (0 to 40), and the RTVue (Optovue, Fremont, CA, USA) recommends ≥45 in signal strength index for macular patterns and ≥35 for retinal scanning, on a scale from 0 to 100^19^.

In the Cirrus HD-OCT 5000, the VD and PD of a local region of tissue according to ETDRS subfields were calculated automatically by the built-in software (AngioPlex, version 10.0). This software provides the VD and PD for the SCP. To calculate the VD and PD, a thresholding algorithm was applied to the SCP en face images to create a binary slab that assigned a value of 1 to each pixel (perfused) or 0 (background). From this slab, a skeletonized slab was created, and the VD and PD were calculated^18^. More specifically, repeated B-scans are acquired from the same tissue location, and motion contrast is created by calculating decorrelation of repeated B-scans. The thresholding algorithm is applied to the obtained unthresholded OCTA B-scan to remove the invalid pixels which are associated with low or noisy OCTA pixels, and then thresholded OCT-B scan image is displayed^20^. Large blood vessels are not a problem. However, smaller sized vessels produce smaller signals. This signal would be more attenuated in lower signal strength, and it might be mixed in background noise. In noise reducing steps, the signal from smaller vessels under a threshold might be masked, leaving no signs of their existence^21^. When angiography images are obtained at high signal strength, the blood flow in small vessels can be correctly detected. However, if angiography analyses are performed on images with low signal strength, all signals are attenuated, making blood flow detection in small sized vessels difficult, so the VD and PD could be underestimated.

Gao *et al*. reported that the VD appeared to be lower in regions where the OCT reflectance signal was weaker because of vitreous opacity or a small pupil^22^. Lei *et al*. performed a cross-sectional study of 42 healthy eyes and 22 retinal diseased eyes for repeatability and reproducibility of OCTA measurements^23^. They concluded that the VD and PD for the SCP showed high levels of repeatability and reproducibility. In addition, they reported that a difference in the SS between scans was positively correlated with a difference in the VD and PD for the whole sample. They observed that if the SS increased by 1 unit, the VD and PD increased by 1.4 mm^−1^ and 0.03, respectively. In the present study, the mean VD, PD, and FAZ metrics increased significantly according to the SS, and if the SS increased by 1 unit, the VD and PD of the total area increased by 1.49 mm^−1^ and 0.037, respectively. The FAZ area also increased by 0.05.

Previous studies have reported normative data for OCTA measurements. However, a recent review of OCTA noted that one of the main problems for analyses of OCTA measurements was that it was very difficult to compare metric calculations across studies because measure protocols and quality control were not standardized. In addition, the details of the quantitative metrics varied among different studies^24^. Iafe *et al*. reported that the FAZ area of patients aged 20–29 years was 0.294 ± 0.112 mm^25^ and Coscas *et al*. reported a mean FAZ area of 0.27 ± 0.10 mm2 in patients aged 20–39 years^7^. The above studies were used with other OCTA instruments; a recent study that used the Cirrus HD-OCT prototype AngioPlex device reported 22.5 ± 0.7 mm^−1^ for the VD, 0.419 ± 0.007 for the PD, and an area of 0.25 ± 0.10 for the FAZ^18^. The mean age of patients in this study was 64.0 ± 7.1 years. A macular 3 × 3 mm angiography scan was performed, with evaluated images having an SS > 6. The results of the previous studies were very different from those of the present study. These differences were especially prominent in the SS 7 and SS 8 groups. Although there were differences in scan protocols among the studies, the FAZ area in the SS 9 and SS 10 groups showed similar results, whereas the areas in the SS 7 and SS 8 groups were only 30–50% of the previously reported values. Considering that all previous studies and the present study included healthy subjects, the SS is a parameter that must be considered in OCTA analyses.

It is difficult to obtain a high SS in patients with ocular media opacity (i.e. corneal opacity, cataract, vitreous opacity, vitreous hemorrhage, etc.) or small pupil. In addition, scan misalignment, non-optimal focusing, and dry eye can cause the change of SS. The above reasons lead to an increase in light scattering, resulting in decreased light penetration in to the retina with the subsequent loss of a signal. The decreased signal might then erroneously appear as an apparent decrease in vascular and perfusion density, but it would be an artifact that results from the loss of signal rather than a real decrease in flow. SS of all subjects varied from 7 to 10, even though they were young and healthy volunteers. Perhaps it is assumed that factors, such as no pupil dilatation, miss alignment, or dry eye may have affected this. If the scan alignment is correct, high signal strength can be obtained without pupil dilatation. However, if there is a scan misalignment or non-optimal focusing, the light will be reflected and scattered from the iris, making it difficult to obtain a sufficient amount of light to penetrate the retina.

All subjects included in this study were young and healthy, and did not have any systemic or ocular abnormalities. In addition, because the analyses were performed at the same age range and all groups that were classified with the SS had the same conditions, the ocular and systemic factors were calibrated. As the SS increased from 7 to 9, the VD, PD, and FAZ metrics also increased significantly. The SS 10 group showed higher OCTA measurements than the SS 9 group, but this difference was not significant for almost all parameters. Because our study analyzed a 6 × 6 mm angiography scan of the macular area, we were not sure how the SS affected the analyses of other areas, such as the optic disc, or other scan protocols, such as the 3 × 3 mm angiography scans. However, because the SS of the present study had a great effect on the macular 6 × 6 mm angiography scans, the OCTA measurements were closely related to the quality of the angiography scan. It is therefore important to examine the SS when reading the OCTA results, and a maximum possible SS is recommended for all angiography scans.

## Methods

This was a prospective cohort study. The study protocol was approved by the institutional review board of the Armed Forces Medical Command (Seongnam, Republic of Korea) and adhered to the tenets of the Declaration of Helsinki. All included subjects met the eligibility criteria and provided written informed consent to participate.

### Subjects

This study initially included 502 eyes from 251 young, healthy volunteers who visited the Armed Forces Capital Hospital for a health screening checkup. All subjects underwent comprehensive ophthalmic examinations including a slit-lamp examination, best-corrected visual acuity (BCVA), intraocular pressure (IOP), axial length, central corneal thickness, and OCTA using the Zeiss Cirrus 5000 (Carl Zeiss Meditec). All subjects underwent a 6 × 6 mm angiography scan in the macular area using the Angioplex from the Zeiss Cirrus 5000, without a pupil dilation.

Exclusion criteria were as follows: a history of systemic disease including hypertension and diabetes; a history of retinal or neuro-ophthalmic disease; glaucoma; a history of ocular trauma; an IOP > 21 mmHg; an OCTA scan with an SS < 7; a BCVA < 20/25; and the presence of a segmentation error in the angiography scan. Finally, 446 eyes from 230 young, healthy subjects were included in this study.

### Optical coherence tomography angiography

The Zeiss HD-OCT 5000 instrument with an AngioPlex OCTA was used to acquire microvasculature images of the macular area. This instrument operated at a central wavelength of 840 nm and a speed of 68,000 A-scans per second. In the 6 × 6 mm scan pattern, there were 350 A-scans in each B-scan along the horizontal dimension and 350 B-scan positions along the vertical dimension. Each B-scan was repeated twice at the same position^26^. The optical microangiography-complex algorithm analyzed the change in complex signals (both phase and intensity changes contained within sequential B-scans obtained at the same position)^27,28^ and then generated en face microvascular images. The vascular images of the SCP, which spanned from the internal limiting membrane to the inner plexiform layer, and deep capillary plexus, which extended from the inner nuclear layer to the outer plexiform layer, were displayed separately. The AngioPlex incorporated the FastTrac retinal tracking technology to minimize motion artifacts.

**Figure 3:**
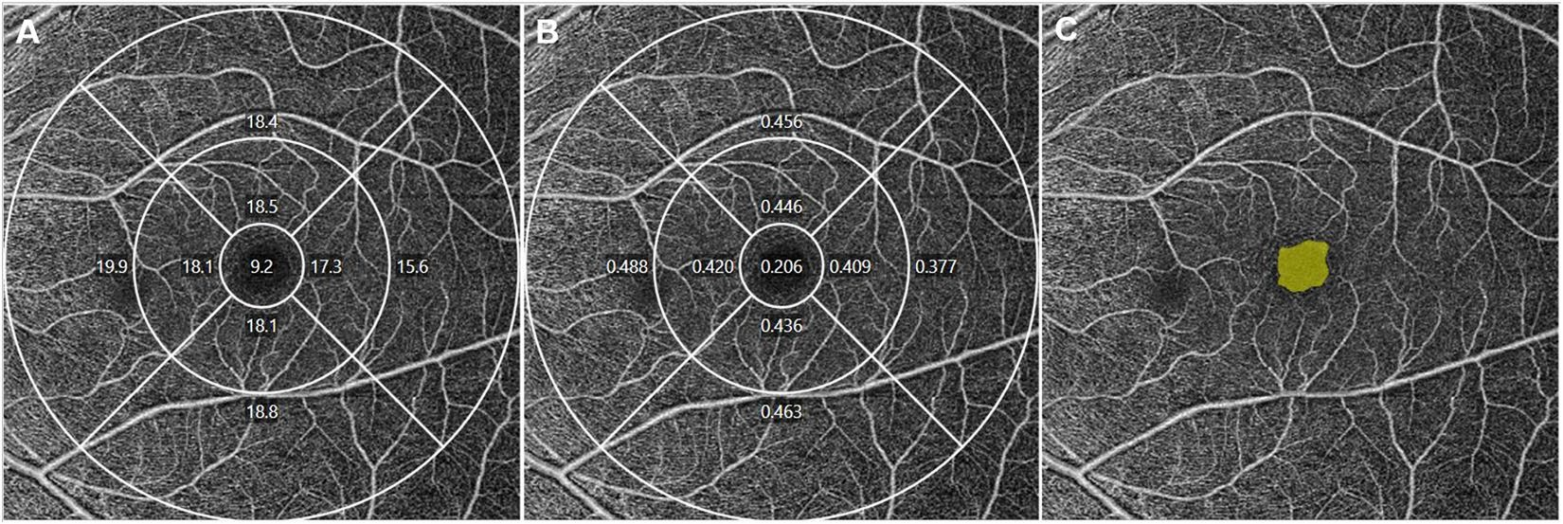
A representative optical coherence tomography angiography (OCTA) image of a 22-year-old healthy male subject in the signal strength 10 group. A vessel density (VD) map of the superficial capillary plexus (SCP) according to the Early Treatment of Diabetic Retinopathy Study (ETDRS) subfields. The VD for the total area was 17.8 mm^−1^ **(A)**. A perfusion density (PD) map of the SCP according to the ETDRS subfields. The PD for the total area was 0.427 **(B)**. The automatically detected foveal avascular zone (FAZ). The area, perimeter, and circularity were 0.28 mm2, 2.09, and 0.81, respectively **(C)**.

All scans were analyzed using the Cirrus OCTA software (AngioPlex software, version 10.0; Carl Zeiss Meditec). VD (defined as the total length of the perfused vasculature per unit area in the region of measurement) and PD (defined as the total area of the perfused vasculature per unit area in the region of measurement) of the SCP according to the Early Treatment of Diabetic Retinopathy Study (ETDRS) subfields were measured automatically (Fig. 3A,B). The area, perimeter, and circularity (defined as 4πA/P, where A was the area and P was the perimeter)^29^ of the FAZ were also measured (Fig. 3C). All scans were reviewed individually by two investigators (H.B.L. and Y.W.K.) for quality assessment (i.e., signal strength, loss of fixation, segmentation error and motion artifacts), and substandard images were excluded. The central foveal thickness (CFT) and retinal nerve fiber layer (RNFL) thickness were also measured using a 512 × 128 macular cube combination scan mode and a 200 × 200 optic disc cube scan mode.

### Statistical analysis

All subjects were divided into four groups according to the SS of the angiography scan (SS 7, SS 8, SS 9, and SS 10). Various clinical factors, including age, sex, BCVA, IOP, axial length, CFT, and average RNFL thickness, were compared among the four groups using the χ^2^ test and one-way analysis of variance (ANOVA) with a post-hoc Bonferroni correction. The VD and PD for the SCP in the macular area were compared among four groups using one-way ANOVA with a post-hoc Bonferroni correction, and FAZ metrics were also compared.

All statistical analyses were performed using SPSS statistical software for Windows, version 18.0 (SPSS, Chicago, IL, USA). Snellen BCVA results were converted to the logarithm of the minimum angle of resolution value (logMAR). Continuous variables were presented as the mean ± standard deviation. Differences were considered significant at p < 0.05.

## Data Availability

Data supporting the findings of the current study are available from the corresponding author on reasonable request.
